# A Systematic Review of Antiamyloidogenic and Metal-Chelating Peptoids: Two Structural Motifs for the Treatment of Alzheimer’s Disease

**DOI:** 10.3390/molecules23020296

**Published:** 2018-01-31

**Authors:** Sherri C. Young

**Affiliations:** Department of Chemistry, Muhlenberg College, 2400 Chew Street, Allentown, PA 18104, USA; sherriyoung@muhlenberg.edu; Tel.: +1-484-664-3271

**Keywords:** Alzheimer’s disease, peptoids, peptidomimetics, beta-amyloid aggregation, beta-amyloid aggregation inhibitors, pharmacokinetics, metal chelators

## Abstract

Alzheimer’s disease (AD) is an incurable form of dementia affecting millions of people worldwide and costing billions of dollars in health care-related payments, making the discovery of a cure a top health, societal, and economic priority. Peptide-based drugs and immunotherapies targeting AD-associated beta-amyloid (Aβ) aggregation have been extensively explored; however, their therapeutic potential is limited by unfavorable pharmacokinetic (PK) properties. Peptoids (*N*-substituted glycine oligomers) are a promising class of peptidomimetics with highly tunable secondary structures and enhanced stabilities and membrane permeabilities. In this review, the biological activities, structures, and physicochemical properties for several amyloid-targeting peptoids will be described. In addition, metal-chelating peptoids with the potential to treat AD will be discussed since there are connections between the dysregulation of certain metals and the amyloid pathway.

## 1. Introduction

Alzheimer’s disease (AD) is an incurable form of dementia afflicting an estimated 5.5 million Americans [1]. By 2050, AD will affect an estimated 13.8 million patients [1] and cost the United States as much as $1.1 trillion [2], representing a growing health, societal, and economic concern. Four of the five AD drugs currently on the market (donepezil, rivastigmine, galantamine, and a donepezil-memantine combination drug) inhibit the enzyme acetylcholinesterase (AChE) in order to mitigate cholinergic dysfunction and subsequent neurodegeneration and memory decline [3,4]. Memantine targets the *N*-methyl-d-aspartate (NMDA) receptor, which, at increased levels, can lead to deterioration in cognitive function [5]. Since marketed AD drugs treat symptoms rather than the underlying disease pathology, there is a dire need for new AD drugs with different mechanisms of action.

From 2002 to 2012, 99.6% of AD drugs that entered clinical trials failed, which is a higher rate of failure than either oncology or cardiovascular drugs [6,7]. Recent high-profile clinical trial failures have slowed progress toward a cure and cast doubt on one of the most widely studied hypotheses for AD pathology, the amyloid hypothesis [8,9]. Challenges surrounding AD drug discovery include mis-diagnosis of clinical trial patients [10,11,12], a lack of reliable biomarkers [10], and an incomplete understanding of the complex and multifaceted disease pathology involving tau hyper-phosphorylation, Aβ aggregation, cholinergic dysfunction, metal dysregulation, inflammation, and oxidative stress [3,6,13,14], which are often interconnected. In addition, AD drugs must penetrate the blood-brain barrier (BBB), further limiting the number of viable therapeutic options [15].

**Figure 1:**
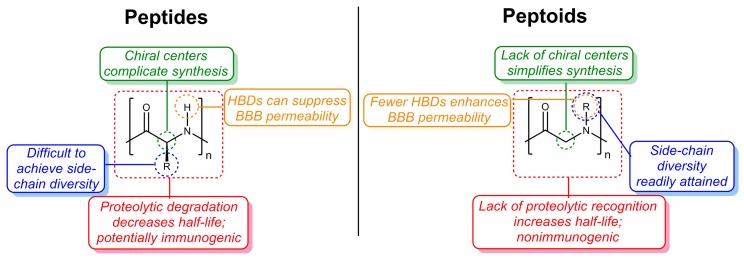
Summary of advantages of peptoids over peptides.

In recent years, peptoids (*N*-substituted glycine oligomers; [16]) have emerged as a promising class of peptidomimetics [17] with a range of properties, including anti-cancer [18] and antibacterial activities [19,20]. Peptoids have several advantages over peptides as drug candidates, including their ease of synthesis via either solid-phase synthesis (SPS; [16,21,22]) or solution-phase methods [23] and their nonimmunogenic nature [24]. Since peptoids are synthesized from primary amine building blocks, of which thousands are commercially available, tremendous side-chain diversity can be readily achieved [25], and their secondary structures are tunable [20]. Furthermore, compared to peptide-based drugs and other biologics, linear and/or cyclic peptoids have more favorable pharmacokinetic (PK) properties, such as longer biological half-lives, due to their unnatural structures, and higher membrane permeabilities, due to a limited number of hydrogen-bond donors (HBDs; Figure 1) [26,27,28,29]. Together, these properties make peptoids viable drug candidates for any disease, but especially for AD and other CNS diseases in which BBB permeability is critical to drug success. 

This review presents the structures, methods used, biological activities, and physicochemical properties for various antiamyloidogenic peptoids. In addition, several metal-chelating peptoids are presented that could prove useful against AD, since there are connections between the dysregulation of certain metal ions and the amyloid pathway [6,30] (vide infra).

The medicinal properties of peptide and peptoid foldamers, including antibacterial properties and their abilities to disrupt protein-protein interactions, have been reviewed [19,20]. The potential of peptoids as AD diagnostic agents, including for the detection of Aβ_1–42_, has been reviewed by Kodadek and Vanderstichele [31] and will not be discussed here.

## 2. Amyloid-Targeting Peptoids

### 2.1. Overview

The amyloid hypothesis, first reported in 1984 [32,33], states that accumulation of Aβ peptide (specifically the more neurotoxic Aβ_1–42_ peptide) oligomers, aggregates, and fibrils plays a role in AD and can trigger a host of effects, including neuroinflammation, taupathy, and neuronal cell death [34]. This hypothesis has replaced the cholinergic hypothesis as the predominant foundation for the development of new AD therapies. Emerging evidence suggests soluble aggregates rather than fibrils are responsible for AD-associated neurotoxicity, which has led to a shift in approach in anti-amyloid drug design [35,36]. Although the importance of the amyloid hypothesis in AD drug discovery has been questioned [8,9], Aβ, and its associated genes, proteins, and pathways, remains the most extensively studied and pursued AD target [34].

**Figure 2:**
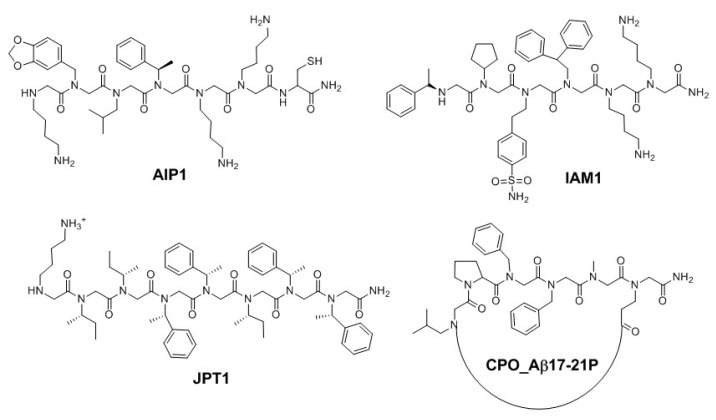
Structures of antiamyloidogenic peptoids AIP1 [40], IAM1 [41,42], JPT1 [43,44,47], and CPO_Aβ17-21P [50]. Protonation states are the same as those shown in the original references.

Several peptide-based drugs have been explored to target AD-associated Aβ aggregation [37]. Due to their low BBB penetrabilities, short half-lives, and low bioavailabilities, many of these compounds are not ideal clinical candidates. Unfortunately, in some cases, attempts to improve bioavailability and stability resulted in decreased inhibition of Aβ aggregation [38]. Amyloid immunotherapies have shown promise, and some have reached clinical trials, but, to date, none have reached the market, in some cases due to adverse effects [39]. Immunotherapies do not penetrate the BBB due to their large size, polarity, and large number of HBDs and hydrogen-bond acceptors (HBAs), further limiting their therapeutic potential [15]. Considering the limitations of current amyloid-targeting therapies, peptoids, which have more favorable PK properties, represent a promising therapeutic class. Section 2 presents various antiamyloidogenic peptoids. Peptoid structures are presented in Figure 2, methods and reported biological activities are in Table 1, and physicochemical properties are summarized in Table 2.

### 2.2. Peptoid-Based Aβ_1–42_-Aggregation Inhibitors

Yang et al. recently discovered the nanomolar Aβ_1–42_ inhibitor amyloid inhibitory peptoid 1 (AIP1; Figure 2). AIP1 increased the lag time to form the critical nucleus for fibril formation and demonstrated in vitro BBB permeability in line with that of previously reported Aβ-aggregation inhibitors (Table 1) [40]. In addition to providing a viable lead for AD, this work presents an approach for the discovery of additional peptoid-based Aβ_1–42_ aggregation inhibitors.

Bezprozvanny and coworkers reported monomer inhibitor of amyloid 1 (IAM1; Figure 2) that selectively suppressed Aβ_1–42_ aggregation and could be used to detect Aβ_1–42_ [41,42]. The authors also investigated the dimer (IAM1)2, which had an even higher Aβ_1–42_ affinity than the monomer and demonstrated neuroprotective properties (Table 1). The peptoid building blocks in this study were chosen to increase structural diversity and water solubility yet allow for Aβ_1–42_-peptoid interactions and suitable BBB penetrability.

### 2.3. Peptoid-Based Aβ_1–40_-Aggregation Inhibitors

Servoss et al. designed peptoids to mimic residues 16–20 of Aβ_1–40_ (KLVFF) [43,44], the hydrophobic region responsible for aggregation [45,46]. One peptoid in particular, JPT1 (Figure 2), adopts a stable polyproline type-I (PPI)-like helical structure, which enhances interactions with Aβ and suppresses peptide aggregation through pi-pi stacking interactions between the peptoid and the phenylalanine residues of the Aβ_1–40_ section. This peptoid suppressed Aβ_1–40_ aggregation in a dose-dependent manner, decreased fibril formation, and decreased the lag time to form fibrils (Table 1). A follow-up study investigated the impact of side-chain chirality and placement on Aβ_1–40_-aggregation suppression [47]. Interestingly, by removing the aromatic side chains or moving the chiral aromatic side chains, inhibition of Aβ aggregation was still maintained; however, the morphology and size of the aggregates changed compared to the original JPT1 analog. The authors proposed that off-pathway aggregates were formed in order to surpass/suppress fibril formation. These studies have enhanced the understanding of Aβ-small molecule interactions and could allow for more effective design of new amyloid therapies. Although the BBB permeabilities of JPT1 and related analogs were not assessed, the lipophilic nature of these Aβ-aggregation inhibitors and the limited number of HBDs could result in suitable BBB permeabilities, although the molecular weights (~1000 Da; Table 2) are outside of the typical range for CNS drugs [48].

Kirshenbaum and Sadowski reported a large library of stable peptide/peptoid hybrids that suppress Aβ_1–40_ fibrillization and oligomerization in vitro and in vivo and mitigate Aβ-mediated NMDA receptor loss (Table 1); these molecules could also be used in AD diagnosis [49]. The approach involved replacing specific amino acid residues of Aβ_1–42_ (or sections thereof) with peptoids or other unnatural amino acids in order to improve PK properties. These peptoids have properties consistent with BBB-permeable molecules (hydrophobic nature, limited number of HBDs, and high plasma stability), making them viable therapeutic candidates. Furthermore, these molecules exhibited suitable BBB penetrabilities, as evidenced by detection in cerebrospinal fluid after intravenous (iv) administration [49].

### 2.4. Peptoids Targeting the Apolipoprotein E4-Aβ Interaction

Apolipoprotein E4 (apoE4), a protein that is a genetic risk factor in late-stage AD, plays a role in Aβ accumulation, plaque formation, and tau pathology. ApoE/apoE4 can interact with specific residues of Aβ to promote aggregation; the apoE4 allele also decreases Aβ brain clearance [34]. As such, the Aβ-apoE interaction has been explored as a target for novel AD therapies. Kwon, Wisniewski, and coworkers discovered CPO_Aβ17-21P (Figure 2) from a small library of cyclic and linear peptoids; this peptoid inhibited apoE4-mediated Aβ aggregation, had a favorable safety profile, improved cognitive function in a transgenic mouse model, and did not trigger a neuroinflammatory response (Table 1) [50]. Furthermore, the fairly low molecular weight (~700 Da; Table 2), hydrophobicity, and cyclic nature of this compound [27,28] could result in favorable PK properties. The BBB permeability of CPO_Aβ17-21P was not explicitly explored, although in vivo efficacy suggests suitable permeability [50].

### 2.5. Comparative Structure-Activity Relationship for Amyloid-Targeting Peptoids

The amyloid-targeting peptoids presented in Section 2 contain aromatic, hydrophobic, and aliphatic residues to facilitate interactions with Aβ_1–40/42_ and/or to improve BBB penetrability [40,42]. In addition, some of these peptoids contain hydrophilic residues (e.g., *N*lys, a lysine-based peptoid residue) to promote water solubility and chiral residues, in particular chiral aromatic residues (e.g., *N*-(*S*)-1-phenylethylglycine (*N*spe)), to promote helicity [44,47].

Aβ_1–42_ aggregation inhibition could be achieved by binding to the N-terminus of Aβ_1–42_ [40], binding to the hydrophobic β-turn-β-sheet region (residues 18–42) of the peptide, or interactions with the C-terminus [42]. Bezprozvanny et al. proposed that the Aβ_1–42_ selectivity of IAM1 was due to interactions between the hydrophobic isoleucine and alanine residues at the C-terminus of the peptide, potentially due to the increased rigidity of this region of Aβ_1–42_ compared to Aβ_1–40_ [42,51]. According to docking studies, the Aβ_1–42_-binding activity of AIP1 could be due to π-π stacking and hydrogen-bonding interactions between the peptoid and Aβ_1–42_ and the peptoid binds to the N-terminus of Aβ_1–42_ [40].

Modulation of Aβ_1–40_ aggregation can occur through π-π stacking interactions between the KLVFF portion of the peptide and aromatic peptoid residues, which may be responsible for the activity of JPT1 [44,47]. Since JPT1 was not assessed as an Aβ_1–42_ inhibitor, it is unclear whether the peptoid would exhibit Aβ_1–40_-selectivity or dual inhibition. The amyloid-targeting peptoids herein exhibit a range of structures, selectivities, and activities to expand the arsenal of potential AD drugs.

## 3. Metal-Chelating Peptoids

### 3.1. Connections between Metal Dysregulation, the Amyloid Pathway, and AD Pathology

**Figure 3:**
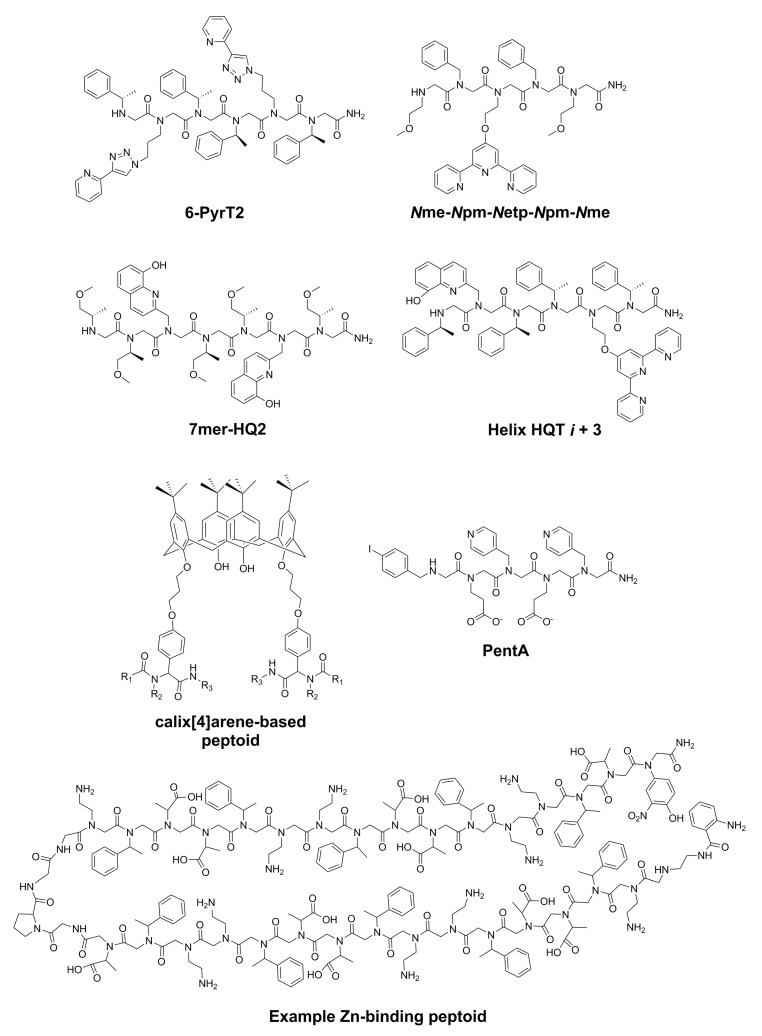
Structures of metal-chelating peptoids 6-PyrT2 [53], *N*me-*N*pm-*N*etp-*N*pm-*N*me [54], 7mer-HQ2 [55], Helix HQT *i* + 3 [58], a calix[4]arene-based peptoid [59], a Zn(II)-binding peptoid [60], and pentA [62]. Protonation states are the same as those shown in the original references.

The dysregulation of Fe, Cu, and Zn plays a documented role in AD, and this dysregulation could be linked to other AD pathologies, including Aβ aggregation and tau hyperphosphorylation [6]. Amyloid plaques contain abnormally high levels of these three metals, and Cu-amyloid complexes have been linked to reactive oxygen species (ROS) generation, mitochondrial dysfunction, and neuronal cell death [6]. In addition, Fe-amyloid complexes can trigger oxidative stress, and an imbalance of all three metal ions can interfere with Aβ degradation [30]. Despite the role of these metals in AD, metal chelators are rarely explored as AD therapies. In addition to addressing metal dysregulation, these drugs could feasibly mediate AD-associated Aβ aggregation, ROS generation, inflammation, and oxidative stress. Developing a relatively nontoxic drug that is selective for the metal ions of interest, can extract metal ions from amyloid plaques, and can penetrate the BBB is a formidable challenge [6]. In Section 3, various metal-chelating peptoids are presented, with a focus on peptoids that bind to AD-associated metals. The structures of these peptoids are presented in Figure 3, methods used and reported biological activities are in Table 3, and physicochemical properties are summarized in Table 4.

### 3.2. Cu(II)-Binding Peptoids

Maayan and coworkers have rationally designed and synthesized multiple classes of metal-binding peptoids, including (1) Cu(II)-binding peptoids with 2-(1*H*-1,2,3-triazol-4-yl)pyridine and 2-(1*H*-1,2,3-triazol-1-ylmethyl)-pyridine side chains as well as *N*spe groups to promote helicity [53]; (2) peptoid 3-, 4-, and 5-mers containing terpyridine, phenathroline, and hydroxyquinoline side chains [54]; and (3) Cu(II)- and Co(II)-binding peptoids with 8-hydroxy-2-quinolinemethyl (HQ) groups for metal binding and hydrophilic (*S*)-1-methoxy-2-propylamine (*N*smp) groups to induce chirality and promote water solubility (Table 3) [55]. Other HQ-containing peptoids were developed to bind Cu(II) and Co(II) [56], and a follow-up study revealed the stabilizing effect of Cu(II) coordination on PP-I-like peptoid helices and the role of peptoid sequence and geometry in folding behavior [57].

Of particular interest are the selective Cu(II)-binding peptoids since this metal plays an important role in AD [6]. For example, helix HQT *i* + 3 (Figure 3) binds Cu(II) selectively when in a mixture of other metal ions such as Zn(II), Fe(III), Co(II), Ni(II), and Mn(II) [58]. This peptoid can also simultaneously bind two different metal ions, such as Cu and Zn, further enhancing the abilities of these molecules to mimic the complex processes of a natural protein. Metal-binding studies revealed that the selectivity and cooperativity are from a combination of helicity and pre-organization of the ligands. The peptoid could also adjust its binding mode (intramolecular vs intermolecular) in response to changes in metal ion concentrations, for example [58].

Varma and coworkers reported a calix[4]arene peptoid tetramer that binds Cu(II) selectively in the presence of various other metal cations (as perchlorates), including Cr(III), Mn(II), Fe(II), Co(II), Cu(II), Zn(II), Hg(II), Ag(I), Pb(II), Cd(II), Na, K, Ca, and Al(III) (Table 3; Figure 3) [59]. One of the peptoids was reported to have high selectivity for Cu(II) based on no change in the observed UV band in the presence of other metal ions. The numbers of HBDs and HBAs as well as the molecular weights of these calix[4]arene derivatives, which range from ~1600 to 2000 Da, could limit the potential of these molecules as CNS drugs (Table 4).

### 3.3. Zn(II)-Binding Peptoids

Zuckermann and coworkers designed heat-resistant two-helix bundles for tight Zn binding to mimic nature [60]. In the presence of the metal ions Mg(II), Mn(II), Ca(II), Ni(II), Co(II), Cu(II), and Cd(II), one of the peptoids exhibited a Zn(II)-binding affinity at least one order of magnitude higher than that of the other metal ions. The Zn affinities for some of the peptoids reported approach that of a Zn-binding protein [60]. This work provides a platform for future screening of peptoid-based metal-binders. The molecular weights (>4000 Da), hydrophilicities, and number of HBDs of these molecules could preclude BBB penetrability, however (Table 4).

### 3.4. Fe(II)-Binding Peptoids

Taillefumier and coworkers reported a series of free and chitosan-bound benzyloxyethyl-based peptoids with both antioxidant and iron-chelating properties for use in food products [61]. Interestingly, binding the peptoids to the chitosan surface resulted in an enhancement of the antioxidant activity of the chitosan itself, although the free peptoids also exhibited some antioxidant properties (Table 3; [61]). Considering the predominant medical use of chitosan films as implants [65,66], the unbound peptoids reported in this study will likely be more useful for AD therapy.

### 3.5. Screening Approaches for the Discovery of Metal-Binding Peptoids

With the capabilities to rapidly generate large compound libraries, high-throughput screening methods to search for peptoids with biologically relevant activities, including metal-binding capabilities, are essential for the discovery of peptoid-based therapies. Kirshenbaum et al. recently reported a bench-top X-ray fluorescence method to screen for metal-binding properties [62], which was validated using a colorimetric assay and ICP-MS. Using this assay, the detection of emission lines can determine metal-binding events. This method requires very small sample sizes and has pmol detection limits, making it an excellent candidate for on-bead analysis of peptoids generated via SPS. Furthermore, the analysis time is on the order of seconds, making this a high-throughput technology. The side chains of these peptoids mimicked histidine, glutamic acid, serine, tyrosine, and tryptophan residues to allow for metal binding, with some non-natural side chains, including pyridylalanine. Metal binding depended on the sequence and structure of the peptoid [62]. Although the authors specifically used this technology to screen for selective Ni(II)-binding peptoids, this approach could be used to search for peptoids that bind metal ions more prominently linked to AD pathology, viz Cu, Fe, and Zn.

^19^F NMR spectroscopy, with aryl fluorides as tags, was used to screen a library of 90 peptoids for Fe- and Cu-binding properties [63,64]. Primary amines containing hydroxyl, imidazole, and carboxyl groups were used as building blocks to attempt to mimic the active site of serine proteases [64]. The metal-binding properties of hits were further investigated using UV-vis titration (Table 3). Selective Cu(II)- and Fe(III)-binding peptoids, some with *K*_D_ values in the micromolar range, were discovered from this screening [64]. Advantages of this approach include the wide availability of the instrumentation and the high sensitivity and large chemical shift dispersion of ^19^F NMR spectroscopy.

### 3.6. Comparative Structure-Activity Relationship for Metal-Chelating Peptoids

As shown in Figure 3, common peptoid ligands for coordination to metals such as Fe and Cu include HQ (e.g., 7mer-HQ2), terpyridine (e.g., Helix HQT *i* + 3), and pyridine derivatives (e.g., PentA) as well as residues to mimic natural amino acids such as histidine or serine, with HQ ligands being the most prevalent among the Cu-binders [55,58]. In addition, chiral aromatic and aliphatic residues were often incorporated to induce helicity, an important property linked to the selectivities observed for some of the metal-binders. Hydrophilic *N*smp groups also promoted water solubility, which could result in more favorable PK properties.

### 3.7. Advantages of Metal-Chelating Peptoids

Many of these peptoids selectively chelate metals and have modifiable secondary structures and PK properties, thereby expanding their therapeutic potential. Furthermore, the ease of peptoid synthesis via the submonomer approach [16,21,22], the tremendous structural diversity possible, and the water solubilities of some of these peptoids, including Maayan’s *N*smp-containing peptoids [55], provide additional advantages. In order to explore the full potential of these molecules as AD therapeutics, their stabilities and BBB penetrabilities should be explored. The metal-chelating peptoids herein have a reasonable balance of hydrophobic and hydrophilic groups; large molecular weights and large numbers of HBDs and HBAs could limit the potential of some of these molecules as CNS drugs (Table 4). Ultimately, the expanded knowledge of peptoid sequence-structure-function relationships will allow for more effective design of peptoid-based metal-chelators for specific applications, such as for the treatment of AD.

## 4. Conclusions

Several important advances have been made in the field of therapeutic peptoids. Peptoid-based Aβ_1–40_- and Aβ_1–42_-aggregation inhibitors were discovered [40,41,42,43,44,47,49,50], some of which are selective and/or BBB permeable [40,41,42]. Various selective metal-chelating peptoids, including those that bind AD-relevant metal ions, have been reported [53,54,55,58,59], and novel screening approaches will allow for the discovery of additional metal-binding peptoids [62,63,64]. In some cases, the metal-binding and peptoid-folding behavior can be controlled based on structure and sequence, thereby approaching the elegance of nature [60]. Based on the connections between Aβ aggregation, AD pathology, and metal dysregulation [6], these molecules represent a promising future direction. Not only do the peptoids presented herein have a range of biological activities relevant to AD, they have the potential to solve many of the PK issues surrounding traditional peptide-based therapeutics [26,27,28,29].

Considering the complex and polyetiological nature of AD, the one-target one-drug paradigm seems insufficient [67], and multifunctional drugs hold promise. Since tremendous structural diversity can be achieved with peptoids, these molecules can be readily modified to incorporate multiple functionalities. For example, a metal-chelating group could be incorporated into an Aβ-aggregation inhibitor, further expanding the potential of peptoid-based AD drugs. 

Out of the 105 potential AD drugs currently in the pipeline [68], none of them are peptoids. With additional advances in the field of peptoids, this should soon change. The biggest hurdles that lie ahead include establishing full PK and in vivo activity profiles, including brain bioavailabilities, for AD-targeting peptoids, continuing to explore the impact of peptoid structure on biological function, and further studying the impact of peptoid sequence on secondary and tertiary structures [69,70]. As the field of biomimetic foldamers continues to advance [71], so will peptoid-based drug design.

## Figures and Tables

**Table 1:** Methods used and bioactivities reported for example antiamyloidogenic peptoids.

Name/Description	Methods Used	Bioactivities	References
AIP1	Combinatorial chemistry was used to synthesize a library of over 4000 peptoids; surface plasmon resonance imaging (SPRi) used to screen peptoid library; thioflavin T (ThT) assay, atomic force microscopy (AFM), ELISA, 3-(4,5-dimethylthiazol-2-yl)-2,5-diphenyltetrazolium bromide (MTT) assay, docking, and circular dichroism (CD) spectroscopy used to investigate antiamyloidogenic activity; in vitro brain microvascular endothelial cell (BMVEC) model used to assess BBB permeability.	*K*_D_ of binding to Aβ_1–42_ is 19.9 nM. Lag time to form critical nucleus increased from 1.78 to 7.18 h with AIP1. Transport ratio of 3.7 ± 0.2% (12 h) measured in BMVEC model.	[40]
IAM1 and (IAM1)2	SPS used to generate an on-bead library containing over 38,000 peptoid analogs; bead-based screening methods, a ThT assay, and an amyloid toxicity assay used to test for Aβ_1–42_-aggregation inhibitory activities.	The Aβ_1–42_ binding affinities for IAM1 and (IAM1)2 are 0.43 ± 0.05 and 0.06 ± 0.04 µM, respectively. IAM1 has higher Aβ_1–42_:Aβ_1–40_ selectivity (9.6-fold vs. 2.1-fold); (IAM1)2 restored viability of neurons to 87% at 100 nM.	[41,42]
JPT1	ThT fluorescence and dot blot analyses used to test antiamyloidogenic properties; fibril morphology investigated using transmission electron microscopy (TEM) and Nile Red spectroscopy; peptoid helicity investigated via CD spectroscopy.	Dose-dependent inhibition of Aβ_1–40_ aggregation was reported (81.2 ± 4.4% at 100 µM of JPT1); fewer Aβ fibrils were formed and the lag time was decreased.	[43,44,47]
Peptoid/peptide hybrids	MTT assay, a ThT aggregation assay, and an oligomerization assay used to assess impact on Aβ_1–40_ oligomerization.	Hybrids suppress Aβ oligomerization; one analog reduced the amount of Aβ_1–40_ oligomers by 61.3%.	[49]
CPO_Aβ17-21P	Linear and cyclic peptoid library synthesized via SPS; SPR, a ThT assay, 3-(4,5-dimethylthiazol-2-yl)-5-(3-carboxymethoxyphenyl)-2-(4-sulfophenyl)-2*H*-tetrazolium) (MTS) cell viability assay, behavioral testing, immunohistochemistry, western blot, and ELISA used to study binding to apoE4-Aβ.	ApoE4-Aβ binding inhibited with a half-maximal inhibitory concentration (IC_50_) of 1.02 nM; transgenic mice exhibited significant cognitive improvement.	[50]

**Table 2:** Physicochemical properties for example antiamyloidogenic peptoids.

Name	Molecular Weight (Da)	HBDs	HBAs ^1^	References
AIP1	970.25	10	20	[40]
IAM1	1037.34	9	18	[41,42]
(IAM1)2	2368.30 ^2^	20	42	[41,42]
JPT1	1130.51	6	18	[43,44,47]
CPO_Aβ17-21P	703.37 ^3^	2	14	[50]

^1^ To determine the number of HBAs, Lipinski’s criteria for hydrogen-bond accepting capacity are estimated by adding up the number of nitrogen and oxygen atoms in the molecule [52]; ^2^ Calculated mass reported in original reference; ^3^ Exact mass reported in original reference.

**Table 3:** Methods used and bioactivities reported for example metal-chelating peptoids.

Name/Description	Methods Used	Bioactivities	References
6-PyrT2 and other Cu(II)-binding peptoids	Peptoids synthesized via microwave-accelerated solid-phase click chemistry; CD spectroscopy and isothermal titration calorimetry (ITC) used to investigate peptoid-Cu(II) binding.	Association constants (*K*_A_) for 6-PyrT2-Cu(II) complex are 3.318 × 10^6^ in methanol and 3.071 × 10^6^ in acetonitrile.	[53]
*N*me-*N*pm-*N*etp-*N*pm-*N*me and other heterocyclic-amine- based peptoids	Peptoid 3-, 4-, and 5-mers containing metal-chelating terpyridine, phenathroline, and HQ side chains synthesized via SPS.	Metal-binding properties were not explored.	[54]
7mer-HQ2 and other Cu(II)- and Co(II)-binding peptoids	Peptoids synthesized via SPS; near-UV CD spectroscopy, UV titration, and EPR used to investigate peptoid-metal binding.	7mer-HQ2 formed a 1:1 complex with Cu(II).	[55]
Helix HQT *i* + 3 and other selective Cu(II)-binding peptoids	Peptoids synthesized via SPS; metal-binding properties studied using UV titration, CD spectroscopy, electron paramagnetic resonance (EPR), and inductively coupled plasma mass spectrometry (ICP-MS) experiments.	*K* for formation of peptoid-Cu complex is 1.03 ± 0.49 × 10^13^ M^−1^; Cu(II) selectivity observed in the presence of over 800× and 670× higher concentrations of Mn(II) and Ni(II), respectively.	[58]
Selective-Cu(II)- binding calix[4]arene peptoid tetramers	Peptoids synthesized using an isocyanide based multi-component reaction (MCR); UV-vis titration used to investigate metal binding.	One of the peptoids binds to Cu(II) selectively in the presence of various metal cations (as perchlorates).	[59]
Selective and tight Zn(II)-binding peptoids	Peptoids synthesized via automated SPS; ethylene glycol bis(2-aminoethyl ether)-*N*,*N*,*N*,*N*-tetraacetic acid (EGTA) competition assay, Förster resonance energy transfer (FRET), CD spectroscopy, and UV-vis spectroscopy used to investigate metal binding.	Some peptoids exhibited Zn-binding affinity at least one order of magnitude higher than that of various metal ions.	[60]
Benzyloxyethyl-based peptoids free and immobilized on a chitosan film with antioxidant and Fe-chelating properties	2,2-Diphenyl-1-picrylhydrazyl (DPPH) and hydroxyl radical procedures were used to assess antioxidant activities; Fe-chelating properties investigated using an EDTA-competition assay.	Two of the free peptoids exhibited concentration- dependent radical scavenging effect of up to 80% at 5 g/L.	[61]
Screening for selective Ni(II)-binding peptoids	SPS used to synthesize peptoids; high-throughput, bench-top X-ray fluorescence screening (with ICP-MS and a colorimetric assay for validation) used to screen peptoids for Ni(II)-binding.	Two of the peptoids bind Ni(II) in the presence of other metal ions.	[62]
Screening for iron- and copper-binding peptoids	Split-pool SPS used to synthesize peptoid library; ^19^F NMR spectroscopy used to screen for metal-binding properties. Results were validated using UV titration.	A 12 nmol detection limit was achieved using a conventional NMR spectrometer; *K*_D_ values of ~27–44 mM measured.	[63,64]

**Table 4:** Physicochemical properties for example metal-chelating peptoids.

Name	Molecular Weight (Da)	HBDs	HBAs ^1^	References
6-PyrT2	1148.39	3	21	[53]
*N*me-*N*pm-*N*etp-*N*pm-*N*me	874.01	3	17	[54]
7mer-HQ2	1091.27	5	24	[55]
Helix HQT *i* + 3	1208.43	4	19	[58]
Calix[4]arene-based peptoids	1613.94–1978.15 ^2^	4–6	14–18	[59]
Zn(II)-binding peptoids	4267.8–4681.2 ^2^	~36–40	~89–93	[60]
Benzyloxyethyl-based peptoids	414.22–512.29	1–2	7–8	[61]
PentA (Ni(II)-binding peptoid)	844.2 ^3^	3	17	[62]

^1^ To determine the number of HBAs, Lipinski’s criteria for hydrogen-bond accepting capacity are estimated by adding up the number of nitrogen and oxygen atoms in the molecule [52]; ^2^ Range of [M + 1] values reported in original reference; ^3^ Calculated *m/z* reported in original reference.
